# Supplementary material for: Are carbon emissions trading and green financial instruments synergistic? -Comprehensive quantitative research based on content analysis
Source: PLoS One. 2024 Mar 7;19(3):e0298601. doi: 10.1371/journal.pone.0298601 (PMC10919601; doi:10.1371/journal.pone.0298601)
Supplement: S1 Data — (ZIP) [file pone.0298601.s001.zip › Data/地级市Z_F工作报告.zip!/║■▒▒/╧╠─■╩╨/└┤╫╘╣1⁄2╓┌║┼í╛┬φ┐╦╩2╛▌═°í┐_2008.docx]

各位代表：

　　 现在，我代表市人民政府向大会作政府工作报告，请予审议，并请市政协各位委员和大会列席人员提出意见。

　　 一、过去四年的工作回顾

　　 2004年以来，在省委、省政府和市委的正确领导下，市政府团结和依靠全市人民，全面贯彻落实科学发展观，求真务实，开拓创新，奋发进取，努力开创咸宁经济社会发展新局面，各项事业攀上新高峰。

　　 经济实力大幅提升。初步测算，2007年，全市生产总值达到280亿元，比2003年增长57.9%，年均增长12.1%；人均生产总值达到10672元，比2003年增加4435元。全社会固定资产投资达到135亿元，是2003年的2.15倍，四年累计完成397.2亿元，年均增长21.1%。社会消费品零售总额达到105亿元，比2003年增长75.2%，年均增长15%。财政全口径总收入达到38.42亿元，比2003年增长2.06倍，年均增长32.3%，其中地方一般预算收入11.1亿元，比2003年增长95%，年均增长18.2%。四年全市建成或基本建成重点项目1724个，湖北咸宁大畈核电和咸宁牌洲湾油气资源勘探项目取得重大进展。赤壁市、咸安区进入全省县域经济20强。“十五”计划顺利实现，“十一五”规划实施良好。

　　 结构调整取得成效。2007年，全市规模以上工业实现增加值79.5亿元，比2003年增长1.86倍，年均增长30%；其中高新技术产业增加值达到9.07亿元，年均增长16%。四年新增规模以上工业企业235家，创建省级名牌产品25个，联乐床垫荣获“中国名牌产品”称号。工业经济效益综合指数由2003的115提高到185，上升70个百分点，创历史新高。第三产业发展加快，增加值达到99亿元，比2003年增长67.1%，年均增长13.7%；其中旅游经济总收入达到15.05亿元，比2003年增长39%。民营经济成份进一步扩大，二、三产业民营经济增加值达到175亿元，占GDP比重达到63%，比2003年提高6.4个百分点。粮食生产从2004年开始扭转了下滑的局面，产量稳定在110万吨以上。特色农业板块基地发展到255万亩，建成市级以上重点龙头企业78家，其中省级17家，绿色、有机、无公害农产品达到68个。全市三次产业结构由2003年的27.3:37.7:35调整为22.5:42.1:35.4。

　　 改革开放步伐加快。全市198户国有工业企业完成新一轮改革改制。率先在全省推进农村综合配套改革，比全省提前一年免征农业税。财政、投融资、行政审批、教育、住房、集体林权制度等一些重点领域和关键环节的改革取得突破。信用环境建设取得明显成效，咸宁市连续四年被评为“A级信用市”。累计完成招商引资项目2007个，引进资金169.2亿元，其中亿元以上项目85个；新增外资企业65家，四年累计直接利用外资1.96亿美元，年均增长12%；红牛饮料、洁丽雅纺织、今麦郎饮品、华新水泥、王老吉饮料、碧桂园、三江航天、武汉新世界、福星惠誉等国内外知名品牌和企业落户咸宁。对外贸易进一步扩大，累计完成出口总额2.28亿美元，年均增长13.3%。积极开展了区域交流与合作，融入武汉城市圈工作取得明显成效。

　　 城乡面貌深刻变化。四年全市用于“三农”的财政支出累计达到11.07亿元，年均增长11.4%；新增通村水泥（沥青）路5402公里；新建户用沼气池4.53万口，同步完成改厨改厕改圈3.16万户；解决了18.06万人的饮水安全问题。加强城乡路网、电网、通信网、广播电视网等基础设施建设，新增公路通车里程7519公里，农村电网改造全面完成，电话普及率提高，广播、电视人口综合覆盖率达到95%以上。市中心城区建设力度加大，淦河城区段两岸整治及休闲带建设、城区道路刷黑、人行道改造、城市污水处理厂和燃气管道等一批重点工程建成或开工建设。全面推进“四城同创”，市区公交系统改革基本完成，成功取缔了城区“麻木”，城市管理水平不断提高。加强县（市）城区和重点镇建设，推进实施“百镇千村”示范工程，城乡面貌明显改观。全市城镇化水平由2003年的41.7%提高到46.3%。

　　 环境质量稳步提高。四年全市用于园林绿化建设的投资1.9亿元，强度创历史之最。市区建成区绿化覆盖率达到45.6%，比2003年提高7.6个百分点；人均公共绿地面积达到9.8平方米，比2003年提高4平方米。狠抓了节能降耗减排，依法关闭了一批污染和高耗能企业。2006至2007年，全市连续2年实现了单位生产总值能耗、化学需氧量和二氧化硫排放“三下降”。中心城区环境空气质量优良率达到97.8%，比2003年提高0.8个百分点。

　　 社会事业全面进步。四年全市用于教育、科技、文化、卫生、体育等方面的财政支出25.83亿元，年均增长10.9%。建立健全了“以县为主”的农村义务教育体制，普通高中教育有了较大发展，职业教育发展加快。四年累计取得省级以上重大科技成果26项，申请专利610件，实施重大科技计划项目21项。公共卫生和疾病预防控制能力加强，新型农村合作医疗实现了对6个县（市、区）全覆盖，参合农民164.01万人，参合率80.2%。群众性文体活动蓬勃开展，社会科学、文学艺术、广播电视、新闻出版事业发展加快。出生人口性别比升高势头得到遏制，低生育水平持续稳定。安全生产管理得到加强，死亡人数连续4年下降。依法治市得到落实，社会政治保持稳定，治安秩序明显好转，恶性案件持续下降。加强和改进信访工作，建立健全了应急管理机制，平安咸宁建设取得实效。民主法制和精神文明建设取得新成果，城乡文明程度进一步提高，公民的法律素质和法制观念明显增强。“双拥”活动深入开展，军政军民关系进一步密切，2007年获“全省双拥模范城市”称号。民族、宗教、对台、外事、侨务、审计、统计、档案、保密、地方志、人防、工会、青年、妇女、儿童、老龄、残疾人等各项工作都取得新的成绩。

　　 人民生活明显改善。全市城镇居民人均可支配收入由2003年的6760元提高到9991元，年均增长10.3%；农民人均纯收入由2003年的2325元提高到3737元，年均增长12.6%。城镇居民人均居住面积34.3平方米，农村居民人均居住面积39平方米，分别比2003年提高7.17和9.34平方米。城乡居民储蓄存款人平5340元，增加2123元，年均增长13.5%。四年累计新增城镇就业12万人，帮助困难群体就业3万人，城镇登记失业率连续四年控制在4.1%以内。全市社会保障支出由2003年的3.11亿元增加到5.68亿元，增长82.6%；参保人数达到86.75万人，其中扩面新增26.45万人，比2003年增长45.8%。四年全市农村极端贫困人口减少2.09万人，低收入贫困人口减少8.3万人。从2004年起，每年为人民群众办好10件实事，并全部完成。

　　 特别是刚刚过去的2007年，全市上下认真贯彻落实市第三次党代会精神，紧紧围绕建设“小康、生态、和谐”咸宁目标，进一步解放思想，抢抓机遇，着力推动又好又快发展，全市经济社会保持了增长较快、结构优化、效益提高、活力增强、民生改善的良好态势，主要经济指标均呈两位数增长，增幅达到或超过全省平均水平。预计全市生产总值增长14.8%，工业增加值增长28%，单位生产总值能耗下降4%，主要污染物排放总量减少2%，全社会固定资产投资增长30.1%，社会消费品零售总额增长19.3%，实际利用外资增长15%，外贸出口增长33.8%，地方一般预算收入增长27.4%，城镇居民人均可支配收入和农民人均纯收入分别增长13.4%和16.3%，城镇新增就业3.84万人，人口自然增长率控制在6‰以内。

　　 各位代表！过去的四年，是我市历史上发展最好最快、人民群众受益最多的四年，通过全市人民的不懈努力，我们圆满完成了市二届人大历次会议确定的目标任务，谱写了咸宁发展史上崭新的篇章。我们这座城市正在发生的深刻变化，极大地增强了咸宁人民的自豪感和责任感，极大地坚定了咸宁人民加快发展的信心和决心，极大地激发了咸宁人民建设美好家园的积极性和创造性。今天的咸宁，正以崭新的风貌，在全面建设小康社会的大道上向前迈进！

　　 回顾过去四年改革和发展的历程，我们深深体会到：

——必须坚持解放思想、与时俱进，始终以创新的思维谋划发展，不断开创各项工作的新局面。四年来，我们坚持以创新的思维深化改革，以务实的态度促进开放，有效化解了前进中存在的困难和问题。认真贯彻落实全市两次加快经济社会发展座谈会精神，开展了声势浩大的思想观念大解放大讨论活动，推进观念创新、制度创新、管理创新；奋力实施“在山上再造一个咸宁”，激发了广大群众创业致富的热情；着力理顺市区管理体制，强化了市区经济的龙头作用；通过创新投融资体制，运用市场化方式盘活城市资产，确保了城市建设的一大批重点项目的顺利实施，有效缓解了政府在投资方面的压力；通过创新招商引资机制和方式，引进了一批发展潜力大、产业关联度高的项目，为咸宁经济的持续发展奠定了基础；通过改进作风、改革体制，着力优化发展环境，着力推进各级各类开发区、工业园区建设，加大产业发展力度，促使我市经济结构发生了重大变化，二、三产业比重不断上升，产业结构日趋合理。

　　 ——必须坚持科学发展、和谐发展，始终高扬加快发展的主旋律，推动咸宁经济社会发展实现新的跨越。四年来，我们深刻理解、全面落实科学发展观，坚持加快发展不动摇，立足自身比较优势，抢抓发展机遇，下大力做好“打基础、管长远”工作，走出了一条符合咸宁实际的发展路子。针对咸宁发展不够的最大实际，组织实施 “项目立市、开放活市、环境兴市、人才强市”战略，进一步强化工业的主导地位，大力推进新型工业化、城镇化和农业产业化，大力推进社会主义新农村建设和发展现代农业，突破性发展旅游业，加快发展县域经济、民营经济，积极推进融入武汉城市圈和承接东部沿海地区产业转移；在加快发展中，更加注重资源节约和环境保护，更加注重社会发展和解决民生问题，促进了全市经济社会进入“全面提速、重点跨越”的新阶段。

　　 ——必须坚持以人为本、关注民生，始终把群众的利益放在首位，最大限度地发挥群众的积极性和创造性。四年来，我们始终坚持发展为了人民、发展依靠人民、发展成果由人民共享，从维护群众根本利益出发，每年坚持不懈地为群众办实事、做好事、解难事，认真解决就业、就学、就医、行路、饮水和食品、药品安全等群众最关心、最直接、最现实的利益问题，坚决纠正在土地征用、城镇居民拆迁、企业重组改制等方面损害群众利益的行为，通过健全社会保障体系，提高城乡低保标准，增加干部职工生活补贴，积极提供公益性事业岗位等多项惠民措施，使发展成果最大限度地惠及了人民群众，广大群众热爱咸宁、建设咸宁的责任感空前增强，在全市范围内形成了人心思干、人心思进的良好局面。

　　 ——必须坚持依法行政、从严治政，始终按照“为民、务实、清廉”的要求，努力塑造政府良好的形象。四年来，我们积极转变政府职能，严格规范行政行为，调整、下放和取消了230项行政审批项目，服务水平和依法办事能力有了新的提高；不断推进决策的科学化、民主化、法制化，认真执行市委的决策部署、市人大及其常委会的决议决定，高度重视人大代表建议、议案和政协委员提案办理工作，四年累计办理670件，办结率、见面率100%，满意率不断提高；深入开展作风和行政效能建设等活动，加强政府系统廉政建设，维护了政府的良好形象；坚持求真务实，把更多精力放在抓落实的具体工作上，开展了干部包保、行风评议、目标管理等工作，不搞虚假的形象工程，不搞劳民伤财的政绩工程，坚决反对形式主义、官僚主义，政府的执行力、公信力和群众满意率明显提高。

　　 各位代表，过去四年的成绩来之不易。这是省委、省政府和市委正确领导，全市人民同心同德、艰苦奋斗的结果；是历届党委﹑政府打下良好基础和市人大﹑市政协监督支持的结果；是各民主党派、工商联、无党派人士，各人民团体，驻咸部队、武警官兵，公安干警，国内外咸宁籍老乡、外来客商和社会各界朋友大力支持的结果。在此，我代表市人民政府，向全市各条战线的广大干部群众，向所有关心和支持咸宁经济社会发展的同志们、朋友们，表示衷心的感谢，并致以崇高的敬意！

　　 必须清醒地看到，咸宁在发展进程中仍然面临不少困难和问题：经济规模和总量偏小，产业结构不合理，发展方式尚未根本改变；对外开放水平不高，影响发展的体制机制障碍依然存在；农业基础仍较脆弱，农民收入稳定增长的长效机制尚未完全建立；社会事业改革和发展相对滞后；公共服务水平和社会保障能力较弱；低收入人群、社会弱势群体的生活还比较困难；投资环境特别是软环境还不能完全适应经济社会快速发展的要求，政府自身改革和建设有待进一步加强，等等。对这些问题，我们一定要高度重视，一定要采取有效措施认真加以解决。

　　 二、今后五年的主要任务

　　 今后五年，是我市在新的历史起点上加速向全面小康社会迈进的重要时期，也是机遇挑战并存，机遇大于挑战的历史机遇期。经济全球化和区域经济一体化深入发展，境外和沿海产业向中西部转移态势不断加强、速度不断加快，全球流动性资金过剩，为我们借力发展，利用好国内外资本、技术、市场，提供了更多的机会和更广阔的空间；国家支持中部地区崛起的具体政策措施正在陆续出台和落实到位，武汉城市圈“五个一体化”进程加快推进，特别是武汉城市圈和长株潭城市群获批全国资源节约型、环境友好型社会建设综合配套改革试验区，咸宁作为武汉城市圈“1+8”成员之一，且处在两大试验区的中心地带，为我市加快改革发展提供了千载难逢的重大历史性机遇。同时，我市工业化、城镇化将进入加速发展期，湖北咸宁大畈核电项目建设和咸宁牌洲湾油气资源勘探开发将加快推进，杭瑞、大广高速公路和城市圈外环公路、武汉至咸宁多条快速通道将陆续开工建设，武广高速铁路客运专线将建成通车，等等，这些将对我市的发展产生重要的带动和支撑作用。只要我们继续发扬艰苦奋斗、敢于拼搏的精神，不断解放思想，抢抓新机遇，争创新优势，开创新局面，咸宁一定能在新的起点上创造新的辉煌！

　　 按照市第三次党代会的总体部署，今后五年政府工作的指导思想是：全面贯彻党的十七大精神，以邓小平理论和“三个代表”重要思想为指导，深入贯彻落实科学发展观，抢抓武汉城市圈综合配套改革试验区机遇，按照建设资源节约型、环境友好型社会的要求，大力推进项目立市、开放活市、环境兴市、人才强市战略，大力推进工业化、市场化、城镇化和农业产业化进程，大力推进体制改革和自主创新，大力推进以改善民生为重点的社会建设，努力实现经济社会又好又快发展，不断提高城市综合实力和竞争力，加快建设小康咸宁、生态咸宁、和谐咸宁。

　　 今后五年我市经济社会发展的主要奋斗目标是：

　　 ——经济发展再上台阶。到2012年，全市生产总值达到600亿元，年均增长14%以上，人均生产总值达到2500美元以上；地方一般预算收入达到27亿元，年均增长20%以上。全社会固定资产投资总额达到410亿元以上，年均增长25%以上；一、二、三产业比重调整为18:45.5:36.5。

　　 ——城市功能明显提升。到2012年，全市城镇化率达到52%。市区建成区面积达到50平方公里，人口规模达到40万人。城市供水、供气、路网等基础设施更加完善，交通、金融、信息、物流、商贸和中介等服务功能明显增强。

　　 ——生态环境持续改善。到2012年，全市森林覆盖率达到56%以上。城市人均公共绿地面积达到12.4平方米以上，污水集中处理率达到70%以上，河流、湖泊水质达标率达到100%，空气质量优良率达到98?%。节能减排完成省下达的目标任务。

　　 ——社会发展更加和谐。教育、文化、卫生等社会事业繁荣发展，创办让人民满意的教育，人人享有基本医疗卫生服务，文化建设实现新提升。全市人口自然增长率控制在6‰以内，居民平均预期寿命达到76岁以上。精神文明和民主法制建设取得新进步，社会安定和谐。

　　 ——人民生活更加殷实。到2012年，城镇居民人均可支配收入达到16000元以上，年均增长10%以上；农民人均纯收入达到6000元以上，年均增长10%以上。城镇职工养老保险覆盖率达到95%，失业保险覆盖率达到80%，城镇登记失业率控制在4.5%以内。

　　 实现上述目标，我们将圆满完成“十一五”规划，开创“十二五”发展良好局面。咸宁现代化进程将发生阶段性、历史性变化。全市经济实力、城市功能、生态环境等将显著提升，咸宁将真正可能成为全市人民感到幸福、引以自豪和令人向往的城市！

　　 为此，我们要着力抓好以下工作：

　　 （一）大力推进经济结构调整和转变发展方式，不断提升工业化水平。进一步强化工业的主导地位，认真研究市场、资源、发展机遇和产业发展趋势，坚定不移地走新型工业化道路，做大规模总量，推进产业结构优化升级。

　　 加快推进新型工业化。按照“做强优势产业、壮大骨干企业、培育经济增长点、提高经济竞争力”的思路，加快推进工业强市。坚持依托园区发展工业，着力打造工业发展平台，加快园区基础设施建设和项目引进工作，实现产业和企业迅速集聚，把园区建设成为经济发展的增长极。突出抓好咸宁经济开发区建设，坚持工业化与城市化并进，加快建设生态工业新城；支持县（市、区）开发区、工业园区建设，鼓励有条件的乡镇办工业小区，使园区成为拉动县域经济发展的“引擎”。到2012年，全市开发区、工业园区的工业增加值在现有基础上翻两番。积极争取湖北咸宁大畈核电项目早日开工，争取实现咸宁牌洲湾油气资源勘探取得实质性突破，规划建设天然气化工新城，支持蒲圻电厂二期、九宫山风电二期和崇阳大湖山风力发电等项目建设，壮大我市能源产业；以红牛、今麦郎、王老吉等知名品牌为核心，加快发展和引进一批食品饮料项目，做大做强食品饮料业；以晨鸣、巨宁等企业为龙头，有序推进森工企业的发展，抓好竹木精深加工，加快发展森工造纸业；培植壮大机电制造、棉麻纺织服装、冶金建材、电子信息、涂附磨具、生物医药等产业，不断提高产业对项目的吸附能力；积极支持赤壁起重机械制造、嘉鱼钢材精深加工、通山石材开发、咸安苎麻纺织服装、通城涂附磨具和崇阳、通城钒产品开发等产业实现集群发展。坚持一手抓龙头，一手抓“成长工程”，着力打造一批龙头型、基地型、品牌型的核心强势企业，力争每年新增规模以上工业企业100家以上，到2012年全市规模以上工业企业达到1000家以上，形成5—8个销售收入达20—30亿元的产业集群。广泛运用信息、节能、新型环保等高新技术和先进适用技术改造提升传统产业，抓好一批高新技术产业化重点项目，促进高新技术产业加快成长壮大。大力实施质量振兴工程和精品名牌战略，加快引进和培育一批创新型企业和名牌产品。坚持“一主三化”方针，促进县域经济、民营经济加快发展，加快培植一批年销售收入过亿元、税收过千万元的县域龙头企业和骨干民营企业，使县域经济实力明显增强，财政状况明显好转，在全省的位次前移。

　　 加快发展现代农业。坚持把“三农”工作作为重中之重，不断增加对农业农村的投入，提高公共财政涉农资金比例，深入推进农业结构调整，大力发展规模农业、特色农业、品牌农业、效益农业，全面推进社会主义新农村建设。稳定粮食播种面积，抓好优质粮食产业工程建设，促进粮食生产稳定发展。突出抓好“在山上再造一个咸宁”工作，加大荒山开发和低产低效林改造力度，加快高产高效林和特色产业基地建设。积极发展多功能农业。大力发展生态观光休闲农业。深入推进农业产业化经营，支持重点农业产业化龙头企业做强做大，突破性发展农产品精深加工业，提高农业产业化水平。力争到2012年，新增省级重点农业产业化龙头企业10家，实现国家级农业产业化龙头企业零的突破。大力发展农业专业合作经济组织，完善农产品流通体系，推进农业标准化生产。支持村级集体经济加快发展。大力发展劳务经济。切实落实各项强农惠农政策，着力提高农民现金收入水平。健全和完善农业科技推广服务体系，加强农业新品种、新技术推广应用，提高农业装备水平。加强农业基础设施建设，突出抓好高产农田建设、病险水库除险加固、中小河流整治以及大中型排灌设施的更新改造。加强农村安全饮水、乡村道路、电网、沼气和信息服务平台建设，进一步改善农村生产生活条件。

　　 加快发展旅游等服务业。进一步整合、深度开发全市旅游资源，深入挖掘咸宁历史文化底蕴，加强旅游基础设施建设，推动旅游产品全面升级。重点建设“温泉生态休闲度假”、“九宫山道佛教避暑观光”、“赤壁陆水三国文化”三大旅游精品，着力打造一批3A、4A、5A级景区。大力发展具有咸宁特色的休闲养生、生态观光旅游和特色乡村游。加快宾馆、酒店和娱乐服务业发展，加大旅游人才的培养力度，提高旅游综合接待能力和服务水平。加强旅游宣传促销，不断完善城市旅游功能，把咸宁建成武汉城市圈乃至华中地区重要的休闲度假和商务会议旅游目的地。到2012年，力争接待游客突破1000万人次，旅游总收入对全市GDP的贡献率达到10%以上。着力发展和壮大商贸、餐饮、市场等传统服务业；支持、引进金融、物流、信息和中介服务业等市场主体，促进现代服务业快速发展；积极培育、引导房地产业健康发展。力争通过五年的努力，全市服务业增加值总量实现倍增、结构明显优化、功能大幅提升，成为优化全市经济结构、实现又好又快发展新的重要支撑。

　　 加快转变经济发展方式。加强自主创新能力建设，为经济结构调整和发展方式转变提供科技进步动力。加快建立以企业为主体、市场为导向、政府推动、产学研相结合的技术创新体系，完善鼓励支持自主创新的政策和激励机制，加快科技创新与服务公共平台建设，鼓励有条件的企业建立研发中心或重点实验室。加大对企业技改项目的支持力度，抓好重点技术攻关。加快企业信息化建设，提高企业运行效率。抓好人力资源开发和人才队伍建设，建立完善激励自主创新的人才评价制度和奖励政策，加强创新型人才培养和使用工作。推进资源节约型、环境友好型社会建设。加快建立科学的节能减排指标体系和监督考核体系，健全节能减排的政策体系和体制机制。积极推进重点行业、重点企业和重点工程的节能减排工作，突出抓好耗能大户、省控排污企业的节能减排，实行“关小上大”，为经济发展腾出环保容量和承载空间。严格控制新建高耗能、高污染项目，加快淘汰落后产能。大力发展循环经济，推进清洁生产和生态化转型，建设一批循环型企业、工业园区和农业生态园区。大力发展环保产业。大力开发利用生物质能。实行最严格的耕地保护制度。坚持用地与造地相结合，科学实施土地整理，实现耕地动态占补平衡。推进硅、钒等资源的合理开发利用。继续抓好一批重点生态工程建设。加强重点流域区域水资源保护和水污染治理。加快县以上城市污水处理设施建设，加快建设垃圾处理场、苎麻脱胶集中处理中心、固体废物处理中心等设施，从源头上防治污染和保护生态。加强地质灾害防治工作。完善资源开发利用、森林生态收益和生态环境恢复补偿机制，加强水利、林业等建设，促进生态恢复。

　　 （二）全面加快以开放促改革促发展步伐，不断提升对内对外开放水平。继续深化改革，全面推进大开放，充分利用国际国内两种资源、两个市场，不断增强经济社会发展的动力和活力。

　　 积极承接产业转移。进一步完善引导产业转移的政策，不断优化发展环境，重点打造承接产业转移的载体，增强对产业转移的集聚和吸纳能力。调整招商引资方向，主动承接国内外特别是“长珠闽”等东部沿海地区和武汉市的产业转移，探索和创新招商思路、招商模式，扩大引资规模，提高引资质量。把“依托优势资源、优惠政策引资”与“产业集群引资”、“创新环境引资”结合起来，探索建立利益共享机制，鼓励联合招商，加快引进一批大项目、好项目、产业集群项目。加强项目库建设，有计划地策划一批大项目、好项目，重点加大对工业、服务业、农产品加工、基础设施和社会事业项目的策划力度。大力实施“回归工程”，鼓励外出务工人员回归创业。通过五年努力，把我市建设成为中部及武汉城市圈内产业转移的重要承接地和协作配套基地、优质农副产品生产加工配送基地和温泉休闲养生度假及会展基地。

　　 加快融入武汉城市圈。按照建设“两型社会”、“五个一体化”和“先行先试”的要求，加快推进与武汉城市圈在观念、体制、产业、市场、基础设施、城乡统筹、环境保护和生态建设等方面的全面对接。明确产业对接的方向和重点，有计划地围绕武汉先进制造业、高新技术产业、现代服务业等，大力开展协作配套；瞄准武汉80万吨乙烯、东风汽车、武钢、富士康和武汉芯片等重大项目建设，策划引进一批配套协作项目。加强与武汉的旅游合作，联手创建圈内无障碍旅游区，着力打造武汉“后花园”。

　　 建立适应大开放的体制机制。在全社会大力营造“崇尚成功、宽容失败、鼓励竞争、激励创新”的人文环境，充分调动广大群众参与改革、大胆探索、大胆实践、敢闯敢试的积极性。以强烈的政治责任心和历史使命感，抓住武汉城市圈综合配套改革试验区机遇，率先在产业配套协作、土地资源管理、城乡统筹、环保、科教合作等方面进行探索和创新，争取在破解区域分割的体制性障碍等问题上走在武汉城市圈前列。深入推进农村各项改革，稳定农村基本经营制度，鼓励农业生产要素向农业企业和种植大户集中，实行适度规模经营；巩固完善农村公益性服务“以钱养事”新机制，努力化解乡村债务，探索建立土地承包经营权流转市场，稳步推进农村集体资产产权制度改革和集体林权制度改革；坚持从优秀村干部中考录乡镇公务员，逐步提高村干部待遇。积极引导企业建立规范的现代企业制度。深化金融体制改革，大力发展城乡股份制商业银行和非银行金融机构，力争实现3—5家企业上市，探索发展适合农村特点的新型金融组织。鼓励、支持和引导个体私营等非公有制经济进入公用事业、基础设施、金融服务以及社会事业等领域，促进非公有制经济加快发展。完善和落实促进全民创业的政策措施，形成有利于各类主体创业的体制机制。深化财政、投资、流通等体制改革。加快现代市场体系建设，大力发展各类要素市场，促进生产要素合理流动。进一步深化行政管理体制各项改革，积极探索大部门管理体制，推进城市相对集中行政处罚权工作。加快推进事业单位分类改革。

　　 大力发展开放型经济。积极扩大外贸出口，重点抓好出口品牌和出口基地建设，支持高新技术产品、优质农副产品、苎麻纺织品、机电产品等优势产品出口，进一步优化出口商品结构，提高出口产品质量、规模和档次。加快实施“走出去”战略，积极推动有条件的企业到境外投资办厂，开展工程承包、服务外包和从事农业综合开发，抓好劳务出口。

　　 （三）科学组织城市规划建设管理与经营，不断提升城镇化水平。坚持规划先行，着力优化城市布局，完善城市功能，加快城市基础设施建设，加大城市管理和经营力度，全面提升城市承载能力，促进城镇化健康发展。

　　 高度重视城市规划工作。进一步强化城市规划的龙头作用。坚持以人为本，立足于可持续发展，着力提高规划的前瞻性、科学性和可操作性。切实加强对规划执行的严格管理，促进规划管理的规范化和法制化。

　　 加快中心城区建设。进一步推进市区一体化，加大市区新区建设和老城区改造力度，围绕规划建设“城北工业新城、城南旅游新城、城中商务政务服务中心”的总体框架布局，以增强城市综合承载和辐射能力为重点，着力加强城市路网、市政、园林等基础设施建设，全面提升城市功能、形象和品位。

　　 切实加强城市管理。深入实施“洁绿亮美”工程，抓好城市交通、市场、经营秩序管理和环境卫生整治，积极稳妥推进“城中村”改造，探索建立城市管理的长效机制，努力争创“国家园林城市”、“国家文明卫生城市”、“国家环境保护模范城市”、“中国优秀旅游城市”。加大城市经营力度，运用市场的机制与办法，管好、盘活、营运城市土地、资产等资源，走“以城养城、以城建城、以城兴城”的路子。

　　 加强县（市）城区和重点镇建设。树立“县当城建”的观念，不断提高县（市）城区建设管理水平。认真贯彻实施《城乡规划法》，探索“以改革为动力、以农民为主体、以规划为导向、以特色产业为支撑”的小城镇发展路子。到2010年，力争每个县（市、区）培育2—3个各具特色的经济强镇，促进农民稳定有序向城镇转移。

　　 （四）着力推进以改善民生为重点的社会建设，不断提升社会和谐水平。在经济发展的基础上，更加注重社会建设，保障和改善民生，努力使人民群众学有所教、劳有所得、病有所医、老有所养、住有所居，建设和谐咸宁。

　　 优先发展教育事业。坚持教育公益性质，加大财政对教育的投入，规范教育收费，健全学生资助制度，保障经济困难家庭、进城务工人员子女平等接受教育。全面实施素质教育，加快普及高中阶段教育，重视学前教育，关心特殊教育，办好人民满意的教育。调整优化教育布局，促进教育均衡发展。大力发展中等职业教育，积极发展高等职业教育和高技能培训。支持咸宁学院、咸宁职业技术学院加强优势学科建设，提高高等教育质量。

　　 千方百计扩大就业。强化政府促进就业职能，多渠道开发就业岗位，大力发展就业容量大的产业，发挥创业带动就业的倍增效应。积极推进城乡就业统筹，做好农村劳动力转移培训工作。完善和落实就业再就业扶持政策，支持下岗失业人员、城镇其他登记失业人员、城镇复员退伍军人及高校毕业生等自主创业。切实解决体制转轨遗留的下岗失业人员再就业问题和改制重组、关闭破产企业的职工安置问题。完善面向所有困难群众的就业援助制度，及时帮助“零”就业家庭解决就业困难。加强职业培训和就业服务体系建设。切实维护劳动者合法权益。

　　 加快建立覆盖城乡居民的社会保障体系。积极推进企业、机关、事业单位基本养老保险制度改革，探索多种形式的农村养老保险办法，解决好失地农民的养老保障问题。全面推进城镇居民基本医疗保险、新型农村合作医疗制度建设。完善城镇和农村居民最低生活保障制度，逐步提高保障水平。完善失业、工伤、生育保险制度。建立城乡居民大病医疗救助制度，逐步提高救助标准。做好优抚安置工作，妥善解决优抚对象的实际困难。抓好防灾减灾救灾工作，安排好受灾群众的生产生活。支持慈善事业发展。加快经济适用房建设步伐，建立健全城市廉租住房制度，有计划、分步骤解决城市低收入家庭住房困难问题。

　　 加快建立基本医疗卫生制度。坚持公共医疗卫生的公益性质，健全农村三级卫生服务网络，资助每个乡镇办好一所卫生院、每个行政村设立一个卫生室，努力让广大农民享有安全、有效、方便、价廉的医疗卫生服务。优化城市医疗卫生资源配置，重点发展社区基本医疗卫生服务，推进惠民医院建设。加强对重大传染病、流行病的预防和监控。加强人口和计划生育工作，完善流动人口计划生育管理服务体系，稳定低生育水平，提高出生人口素质，综合治理出生人口性别比偏高问题。

　　 加强文化建设。坚持社会主义先进文化的前进方向，深化文化体制改革，推动文化创新，提高文化软实力。大力发展公益性文化事业，加强社区和乡村文化设施建设，构建与经济社会发展水平相适应的覆盖全市的公共文化服务体系。完成市图书馆、博物馆、科技馆、群艺馆、大剧院及城市公园等文化设施建设。支持文化产业加快发展，培育一批文化产业骨干企业和新的文化业态，繁荣文化市场。加强网络文化建设和管理。深度挖掘地方特色文化资源，努力建成一批体现时代特征、咸宁特色、群众喜闻乐见的文化项目和文化品牌。加强城乡基层体育设施建设，广泛开展全民健身活动，提高体育竞技水平。繁荣发展哲学社会科学、新闻出版、广播电视、文学艺术事业。加强社会主义核心价值体系和思想道德建设，深入开展群众性精神文明创建活动，努力在全社会形成共同的理想信念和良好风尚。加强青少年思想道德教育工作，为青少年健康成长创造良好的社会环境。

　　 加强民主政治建设。健全民主制度，丰富民主形式，拓宽民主渠道，发展基层民主，保障人民享有更多更切实的民主权利。坚持依法治市，促进公正、民主、文明执法。深入开展法制宣传教育，形成自觉学法守法用法的社会氛围。深入推进“平安咸宁”建设，加强社会治安综合治理，加大打黑除恶力度，依法严厉打击各种违法犯罪活动，坚决扫除“黄赌毒”等社会丑恶现象。加强国家安全工作。关心和支持驻咸解放军、武警部队建设，深入开展国防教育和双拥共建活动，健全国防动员体系，抓好人民防空和民兵预备役工作，巩固军政军民团结。

　　 努力维护社会稳定。严格执行安全生产责任制，加大安全生产事故责任追究和处罚力度，防止重特大事故发生。加强卫生监督综合执法和卫生监督体系建设，强化食品、药品、餐饮卫生监管，保障人民群众健康安全。加强价格监测、预警和监管，抓好“米袋子”、“菜篮子”建设，努力保持居民消费价格基本稳定。进一步整顿和规范市场经济秩序，依法严厉打击制假售假、商业欺诈等行为。健全政府、企业和个人信用体系，完善信用监督和失信惩戒机制。完善预警和应急救援机制，提高保障公共安全和处置突发事件的能力。进一步完善社会利益协调和社会矛盾纠纷调处机制，做好人民群众来信来访工作。加大对老区、库区和贫困地区的扶持力度。认真做好民族、宗教、外事、侨务、对台等工作。

　　 （五）切实加强政府自身改革和建设，不断提升“科学、民主、依法”行政水平。深化行政管理体制改革，推进制度和管理创新，不断优化投资环境，强化科学发展的行政体制保障，全面建设“创新、亲民、务实、廉洁”责任政府，提高政府公信力、执行力和群众满意率。

　　 加快转变政府职能。把学习作为提高素质、做好工作的第一要求和基本途径，增强贯彻落实科学发展观的自觉性和坚定性。切实把政府管理经济工作的着力点放在为各类市场主体服务和创造良好环境上。依法界定政府职责，优化政府机构设置，强化社会管理和公共服务职能，加大对公益性、基础性领域的投入，增强政府提供公共产品和服务的能力。

　　 全面推进依法行政。健全行政决策机制，完善重大事项集体决策、专家咨询、社会公示与听证、决策评估等制度，推进决策的民主化、科学化。建立健全决策反馈纠偏机制和决策失误追究制度。深入贯彻落实《全面推进依法行政实施纲要》和《行政许可法》，严格按照法定权限和程序行使权力，不断提高依法行政水平。认真落实市委决策、人大及其常委会的决议决定，坚持向人大及其常委会报告工作、向政协通报情况制度，自觉接受人大及其常委会的法律监督，接受人民政协的民主监督。认真办理人大代表议案、建议和政协委员提案。积极探索综合执法改革，健全和落实行政执法责任制、行政执法过错责任追究制。

　　 加强政府效能建设。大力倡导“干事文化”，进一步转变作风，努力形成“八个方面”的良好风气。牢固树立“服务第一”的观念，让职责、权力服从服务于发展的需要，让规矩、程序服从服务于效率的需要，让评价、结论服从服务于最后的成果业绩。继续深化行政审批制度改革，完善行政审批方式，加强审批和服务载体规范化建设，努力为企业提供“保姆式”服务。推行电子政务，创新行政管理模式，优化政府管理流程，进一步提高办事效率。加大政务公开力度，继续完善信息公开、公开办事、新闻发言人等制度。完善目标管理责任制和绩效考核指标体系，推进政府绩效管理。严格执行行政问责制，严肃查处“不作为”、“乱作为”、失职渎职行为，着力提高政府执行力，确保政令畅通。

　　 推进政府廉政建设。严格执行党风廉政建设责任制，加强对政府工作人员的严格要求、严格教育、严格管理、严格监督。健全完善教育、制度、监督并重的惩治和预防腐败体系。加强审计监督和行政监察。坚决纠正损害群众利益的各种不正之风，切实解决群众反映强烈的问题，从严惩治商业贿赂等腐败行为。大力倡导勤俭节约、勤俭办一切事情，坚决反对奢侈浪费。

　　 三、2008年的主要工作

　　 2008年是全面贯彻落实党的十七大作出的战略部署的第一年，是实施“十一五”规划承上启下的一年，也是新一届政府的开局之年。同时，我们将迎来北京奥运会的举办和改革开放30周年。做好今年的工作，意义十分重大。我们一定要认真贯彻党的十七大、中央和全省经济工作会议精神，牢固树立“稳中求进、好字优先”和“好中求快、紧中求活”的思想，全面落实科学发展观，积极应对武汉城市圈获批国家综合配套改革试验区的机遇，坚持用改革的办法破解难题，用市场的办法推动发展，不断开创经济社会又好又快发展新局面。

　　 今年全市经济社会发展的主要预期目标是：生产总值增长15%，规模以上工业增加值增长25%以上，全社会固定资产投资增长25%以上，社会消费品零售总额增长15%以上，出口总额增长15%以上，外商直接投资增长15%以上，地方一般预算收入增长20%以上，城镇居民人均可支配收入增长10%以上，农民人均纯收入增长10%以上，居民消费价格涨幅控制在4.8%以内，城镇登记失业率控制在4.5%以内，人口自然增长率控制在6‰以内，单位生产总值综合能耗下降4.4%，化学需氧量和二氧化硫排放完成省下达的减排任务。

　　 为此，我们要重点抓好以下几项工作：

　　 （一）加大工业发展力度，推进新型工业化。按照“工业园区化、园区产业化”的思路，大力发展园区经济。重点抓好咸宁经济开发区建设，完成长江产业园一期“七通一平”，启动二期基础设施建设，做好迁村腾地工作，建成产业孵化园，强化园区招商引资，确保建成项目20个，招商引资到位资金30亿元，新增产值8亿元，税收增长1倍。支持县（市、区）开发区、工业园区加快建设，支持崇阳、通山、咸安做好省级经济开发区的报批工作。集中力量抓好一批重大项目建设。积极推进湖北咸宁大畈核电、咸宁牌州湾油气资源勘探项目前期工作。加快推进红牛饮料二期、电子信息产业园、蒲圻电厂二期、富士康配套产业园、华新水泥二期、九宫山风电二期以及武汉爱帝、佐尔美、今晨、元田集团等一批重点工业项目建设。实现今麦郎二期、汇美达三期、奕东电子三期、天化麻业新增16万锭生产线、嘉鱼金嘉木业、通城华夏建龙一期、崇阳众望食品新线、通山德行玻璃等项目建成投产。策划引进山东晨鸣纸业50万吨林纸一体化等项目落户咸宁。支持全市20家高新技术产业企业的新产品开发、技术攻关和产业化项目。推动盘活盘强蒲纺工业园。继续抓好机电制造、食品饮料、冶金建材、棉麻纺织、涂附磨具等产业集群发展。制定实施推动企业上市的政策措施，扶持优势企业做好上市前的准备工作，力争实现上市公司零的突破。继续抓好“中小企业成长工程”。着力推进“四个一批”企业的培育，力争新增规模以上工业企业100家以上，新增1—2家企业进入全省“三个三工程”。重点扶持玉立砂带、三环汽车方向机等企业争创中国名牌和驰名商标，力争新增1—2个中国名牌产品。加强工业生产调度，切实解决企业生产经营中的困难和问题，支持帮助困难企业扭亏增盈。

　　 （二）扎实做好“三农”工作，发展现代农业。继续抓好优质粮食产业工程，力争粮食总产稳定在110万吨以上。支持通城、崇阳规划建设生猪养殖大县，支持咸安规划建设畜禽养殖大县（区），支持嘉鱼进入全省水产十强县。积极推进“在山上再造一个咸宁”，重点建设好崇阳、咸安、通山3个10万亩水果产业板块，咸安、赤壁、崇阳3个10万亩泡桐基地，新建特色农业基地面积30万亩；加大荒山开发和低产低效林改造力度，完成25万亩林业开发任务。推进农业标准化生产，新增无公害农产品、绿色食品和有机食品品牌20个。制定农产品加工产业发展规划。重点扶持年销售收入过5亿元的农业产业化龙头企业，发展年产值过50亿元的特色产业，新增投资过2000万元以上农产品加工企业6—10家，着力培植国家级农业产业化龙头企业。组织专业科技人员对农业产业化龙头企业、产业化基地和重大科技项目进行帮扶，推广一批农业增产增效新品种和先进适用技术，提高农机补贴标准。抓好农田水利基本建设，加大病险水库、低产田改造力度。推进农村山林地、冬闲田等土地流转工作。继续抓好新农村建设示范村建设，组织开展以村庄村容整洁为重点的农村“清洁家园行动”试点。加强农村商贸、文化等设施建设。

　　 （三）大力发展旅游业，提升第三产业发展水平。制定完成《咸宁旅游总体规划》。继续抓好九宫山、赤壁古战场、温泉谷、潜山国家森林公园、陆水湖等重点景区提档升级。加快推进碧桂园、三江航天、通圣太乙休闲度假村、赤壁龙佑温泉二期、嘉鱼三湖温泉、通山隐水洞等重点旅游项目建设。启动温泉至澄水洞、温泉至星星竹海、旅游新城外环干道等旅游公路建设，着力打造咸宁旅游经济圈。加快咸宁温泉游客集散中心、文化娱乐一条街、餐饮美食一条街等建设。重点抓好赤壁、贺胜2个旅游特色镇建设，建设田野、沧湖生态示范园，争创2个国家级农业旅游示范点。继续抓好“中国优秀旅游城市”创建工作。加大旅游宣传力度，精心组织首届“咸宁温泉文化旅游节”，支持办好“三国赤壁文化旅游节”、“九宫山避暑节”等旅游节庆活动。坚持用现代经营手段和信息技术改造提升传统服务业，大力发展商贸、餐饮、休闲娱乐业，加快市建材批发市场、农产品批发市场等专业市场建设，促进有形市场与电子商务有机结合。加大市场开拓力度，新增5—6个地产品品牌进入大超市。继续抓好“万村千乡市场工程”建设，新建标准化农家店410家。

　　 （四）加强资源节约，保护生态环境。实行严格的节能减排工作责任制和问责制，确保节能减排目标任务全面完成。继续抓好全市37家重点能耗企业和省定重点企业的节能降耗工作。推广先进节能技术，大力发展循环经济，全面推行清洁生产，加快实施工业园区生态化改造。深入开展节能减排全民行动。严格实施环境影响评价制度，严格控制新污染。实现咸宁市中心城区和赤壁市2个污水处理厂项目按期建成并投入运营，启动实施5个县（区）城市污水处理厂规划和建设。加快推进苎麻脱胶集中处理中心建设。加强陆水湖、富水湖、西凉湖、斧头湖等湖泊的保护和合理开发。继续推进天然林保护、退耕还林、长江防护林、水土流失治理等生态工程。加强土地计划管理，优先保障重点项目建设用地，落实经营性用地招拍挂出让制度，优化土地资源配置。抓好闲置土地、闲置厂房的开发再利用，提高土地利用集约化程度。

　　 （五）抓好中心城区建设，加快城镇化进程。继续做好咸宁城市总体规划的修编、近期建设规划编制及土地利用总体规划修编工作。继续加大投入力度，进一步抓好城市路网、市政等基础设施建设和城市功能完善。加快武广高速铁路客运咸宁站、咸宁中心客运站、城区南环路及咸（宁）甘（棠）一级道路建设，完成潜山路、滨河西路、滨河东路、桂花路等4条道路刷黑，启动9条人行道及其排水改造工程建设，新（续）建4个广场、公园建设项目。加快推进城市污水处理、垃圾处理、王英水库引水等城市配套设施建设。抓好行道树、绿化带建设，新建4个街心小游园，新增城市公共绿地面积12万平方米。加大旧城区改造和 “城中村”综合改造力度。新增一批公交线路。启动实施城市灯光环境建设工程。积极推进“四城同创”，大力实施环境清洁工程，加大“两违”和“脏、乱、差”专项整治力度，探索建立城市管理综合执法体系。加强城市土地市场的管理，盘活城市存量资产。继续抓好5个县（市）城区建设，积极协调解决县（市）城区及小城镇建设中的实际问题。坚持产业兴镇（村），推进20个重点镇和50个“百镇千村”示范工程建设。

　　 （六）全面深化改革，协调对内对外开放。进一步完善农村公益性服务和农村义务教育经费保障机制，提高“以钱养事”标准和农村中小学预算内公用经费补贴标准。深入推进集体林权制度改革，确保今年完成确权发证任务。加快培育产权、资本、技术、信息和劳动力等要素市场，加速融资方式创新和金融市场的发育，破解城建、中小企业和农业等融资难题。积极引进股份制商业银行及非银行金融机构来我市设立分支机构或开展业务。推进农村信用社改革，扩大乡村银行试点。抓紧解决国企改革遗留问题，建立规范的现代企业制度。积极推进非工领域企业改革。加快推进党政机关所办企业和从事企业经营管理的事业单位的脱钩改制工作。加强企业信用体系建设。大力加强财源建设，加强非税收入管理，深化财政管理体制改革，加快建立完善公共财政体系。加快推进社会管理体制改革，完善公共服务体系。精心组织参加中博会、鄂港经贸洽谈会、厦门投洽会、深圳高交会等重大招商活动，巩固小分队招商，做好委托商会招商工作。积极主动承接东部沿海地区产业转移。进一步加大“回归工程”实施力度。加强项目库建设，认真做好项目策划工作，切实抓好招商信息、项目、合同的跟踪落实。扩大地产品出口，推进机电产品、纺织品、工装系列、医用敷料出口基地建设。积极发展出口企业，开拓出口市场。进一步扩大区域经济合作。科学制定融入武汉城市圈综合配套改革试验区的实施方案，积极推进武汉经济开发区、东湖高新技术开发区在咸宁建设“园外园”，力争在与武汉市的产业对接和一体化建设上有实质性进展。推动金口至嘉鱼一级公路建设，做好贺（胜）纸（坊）公路改造及新辟一条快速通道的准备工作。

　　 （七）着力改善民生，促进社会和谐。进一步完善义务教育经费保障机制。多渠道化解“普九”债务。加快“义务教育阶段农村寄宿制学校建设工程”和“农村中小学改水、改厕”项目建设。完成市区教育布局调整，整合教育资源，在新城区启动新建一所“九年一贯制”学校，抓好泉塘小学改造工程。千方百计扩大就业。继续抓好“4050”下岗失业人员和零就业家庭就业援助工作，加大小额担保贷款力度，鼓励下岗职工自谋职业、自主创业。进一步扩大社会保险覆盖面，新增基本养老、基本医疗、失业、工伤和生育保险参保人员3.75万人。健全社会救助体系，完善城乡居民最低生活保障制度。在6个县（市、区）的中心城区分步推进失地农民参加养老保险工作，开展农村养老保险试点工作。争取我市列为全国第二批城镇居民医疗保险试点城市，启动城镇居民医疗保险试点工作。抓好市中心医院迁建工作，办好惠民医院，启动咸宁市老年公寓、流浪未成年人救助保护中心、儿童福利中心建设。加强对艾滋病、结核病、血吸虫病等重大传染病、流行病的预防和监控。稳步推进“药品直供”、“医药分开”试点。做好人口和计划生育工作，抓好县、乡两级计划生育服务机构的建设。启动市博物馆、妇女儿童活动中心、青少年活动中心建设，完成市体育中心改造、金叶体育馆整修工程。整顿和规范市场经济秩序，加强食品、药品安全监管，维护消费者合法权益，切实改善消费环境。加强安全生产监督管理。落实信访工作责任制，防范、化解社会矛盾。

　　 （八）推进管理创新，提高行政效能。深入开展“行政效能建设年”活动。继续深化行政审批制度改革，进一步减少和规范行政审批事项和审批环节，在市行政服务中心建立规范的行政审批服务窗口，按照“应进必进”的原则，所有行政审批事项一律进中心。全面实行重大项目全程办事代理制。认真抓好中介机构与职能部门利益脱钩。严格执行“收支两条线规定”，推进收支脱钩、罚缴分离。进一步抓好行政事业性收费清理，进一步减少收费项目、降低收费标准。完善行政投诉、行政效能电子监察平台，加强对行政效能的全过程监督。进一步抓好土地储备交易中心、招投标中心、产权交易中心、政府采购中心、经济发展环境投诉中心建设。加大政务信息公开力度，办好政府门户网站。深入实施行政执法责任制，认真落实行政过错责任追究制度。加强政务督查，推进各项工作的落实。

　　 各位代表，努力为人民群众办实事，是各级政府应尽的职责。新一届政府将继续坚持为民办实事制度。今年，在广泛征集意见的基础上，本着“普惠、解难、量力、当年”的原则，继续办好10件实事。
